# The Cell Origin and Role of Osteoclastogenesis and Osteoblastogenesis in Vascular Calcification

**DOI:** 10.3389/fcvm.2021.639740

**Published:** 2021-04-23

**Authors:** Wenhong Jiang, Zhanman Zhang, Yaodong Li, Chuanzhen Chen, Han Yang, Qiuning Lin, Ming Hu, Xiao Qin

**Affiliations:** Department of Vascular Surgery, The First Affiliated Hospital of Guangxi Medical University, Nanning, China

**Keywords:** vascular calcification, osteoblast-like cells, osteoclast-like cells, origin, reversibe, cell therapy

## Abstract

Arterial calcification refers to the abnormal deposition of calcium salts in the arterial wall, which results in vessel lumen stenosis and vascular remodeling. Studies increasingly show that arterial calcification is a cell mediated, reversible and active regulated process similar to physiological bone mineralization. The osteoblasts and chondrocytes-like cells are present in large numbers in the calcified lesions, and express osteogenic transcription factor and bone matrix proteins that are known to initiate and promote arterial calcification. In addition, osteoclast-like cells have also been detected in calcified arterial walls wherein they possibly inhibit vascular calcification, similar to the catabolic process of bone mineral resorption. Therefore, tilting the balance between osteoblast-like and osteoclast-like cells to the latter maybe a promising therapeutic strategy against vascular calcification. In this review, we have summarized the current findings on the origin and functions of osteoblast-like and osteoclast-like cells in the development and progression of vascular progression, and explored novel therapeutic possibilities.

## Introduction

Vascular calcification, or the pathological accumulation of calcium phosphate crystals in the intimal and medial layers of vessel walls, is the pathological basis of many cardiovascular diseases (1, 2). It decreases blood vessel compliance and leads to lumen stenosis, eventually aggravating the cardiovascular symptoms. More than 60% of the middle-aged and elderly individuals in the 40–75 years age group, and more than 70% of patients undergoing chronic dialysis exhibit aortic calcification (3, 4). Furthermore, the prevalence of vascular calcification reaches as high as 80% in the patients with atherosclerosis, aged 80 and above (5). Although the mechanisms underlying vascular calcification have been elucidated to a large extent, there is still a dearth of effective pharmacological therapies. Thus, there is an urgent need to further clarify its molecular and cellular basis.

Vascular calcification was initially recognized as a passive and degenerative pathological process. However, recent findings indicate a cell mediated, active and reversible process that is similar to physiological bone mineralization (6). For instance, chondroblast-like cells, osteoblast-like cells, and even complete lamellar bone and regenerated bone marrow have been observed in the walls of calcified arteries (7–9). Furthermore, several bone-specific transcription factors and bone matrix proteins are overexpressed during vascular calcification, and their inhibition blocks osteoblast-like transdifferentiation and calcification (10, 11). In addition, calcium salts released from osteoporotic bones are deposited in the vascular lesions, which further points to a crosstalk between the osteogenic and vascular systems (12–15). Therefore, researchers are increasingly recognizing a novel bone-vascular axis in the pathogenesis of vascular calcification (16, 17), wherein osteoblast-like cells play a central role. Although there are no osteoblast-like cells in the normal arterial walls, studies show that vascular smooth muscle cells (VSMCs) can differentiate into chondroblast/osteoblast-like cells in the presence of high calcium levels (18, 19). In addition, endothelial cells, fibroblasts, pericytes, mesenchymal stem cells, and progenitor cells can also transdifferentiate into osteoblast-like cells (20–24). Therefore, the exact origin of osteoblast-like cells in the calcified arterial walls and the subsequent phenotypic remodeling are largely unknown.

Interestingly, vascular calcification may be reversible. For instance, calcification scores of both coronary and carotid artery are significantly declined in several patients with end-stage renal disease after they undergo subtotal parathyroidectomy (25). Additionally, aortic calcification induced in uremic rats with calcitriol and high phosphorus diet can be partially resolved when reducing phosphorus intake or using calcimimetics or phosphate binders, which may be involved in increase of mineral phagocytes and urinary calcium excretion (26–29). Rat medial elastocalcinosis that is induced by warfarin-rich diet feeding would also be inhibited when they are feeding with vitamin K-rich diet, with content in the aorta decreased by 50% (30). Interesting, aortic mineral loss of rat with medial elastocalcinosis is associated with activation of carbonic anhydrase IV that plays an important role in bone resorption, suggesting vascular mineral loss has a similar mechanism with bone resorption (31). Medial artery calcification is induced with calcitriol. It will regress rapidly, displaying a 75% decline of aortic calcium and phosphorus in 9 weeks. Furthermore, the reversibility of calcitriol induced vascular calcification may be mediated by macrophages (32). Apart from these, the phenotype of osteoblast-like VSMCs can be also reversed. It is reported that the initial deposition of hydroxyapatite-like mineral in medical arterial calcification occurs on degraded elastin first and that causes VSMCs transdifferentiation into osteogenic phenotype expressing bone related proteins and contribute to VSMCs calcification *in vitro*, but these VSMCs will return to original phenotype of VSMC after calcified conditions are removed (33). These researches indicate vascular calcification is reversible that is possibly involved in mineral loss similar to bone resorption, but the reversal process is still largely unknown.

Under physiological conditions, the osteoblasts and osteoclasts maintain the balance between bone calcium absorption and resorption, which is essential for proper bone mineralization (34, 35). There is evidence showing the involvement of osteoclast-like cells in vascular calcification (36). Ge et al. reported the increased expression of TRAP, which is a osteoclast phenotypic marker in the calcific vessels from hypertensive patients (37). Qiao et al. also identify osteoclast-like cells are present in carotid artery atheromatous lesions containing calcified plaque based on cell morphology, TRAP-positivity, and osteoclast-associated marker genes. Furthermore, they think osteoclast-like cells absorb deposited minerals preventing vascular calcification and counteract the effects of osteoblasts (38). In addition, osteoclasts cultured *in vitro* with calcified elastin reduced the extent of calcification and restored the structural integrity of elastin, indicating that these cells have an inhibitory role in vascular calcification as well (39, 40). Therefore, enhancing the proportion of osteoclast-like cells in the calcified arteries might be a suitable therapeutic intervention. However, the origin, phenotypic transformation and functions of the osteoclast-like cells during vascular calcification also remain to be elucidated.

Therefore, we have summarized the current knowledge regarding the osteoblast/osteoclast-like cells in vascular calcification, and discussed their potential as therapeutic targets.

## Molecular Mechanism Underlying Osteoblast-Like Cells-Mediated Vascular Calcification

Studies show that the initiation and progression of vascular calcification likely involve both active and passive mechanisms (41–43). Passive calcification depends on the natural affinity of elastin and collagen in the extracellular matrix (ECM) for calcium ions. These proteins bind to calcium ions and increase their deposition in the ECM, resulting in extracellular calcium and phosphorus imbalance. The positively charged calcium ions adsorb the negatively charged phosphoric acid or carbonate ions to form amorphous calcium salt, which continuously adsorbs more mineral ions to form large mineral deposits and eventually lead to vascular calcification (41, 44, 45). Active vascular calcification resembles physiological bone mineralization. The cells in the vessel walls differentiate or transdifferentiate into chondroblast/osteoblast-like cells in the pro-calcific milieu of diabetes, chronic kidney disease, aging, and atherosclerosis. The osteoblast-like cells express osteogenic transcription factors and genes, such as runt-related transcription factor 2 (Runx2), osterix, Msh homeobox (Msx), SRY-box transcription factor 9 (SOX9), and alkaline phosphatase (ALP), bone sialoprotein (BSP) and osteocalcin (OC), all of which play a central role in bone mineralization. Knocking down RUNX2, osterix, SOX9, MSX1, or MSX2 suppressed osteogenic differentiation and subsequent vascular calcification (10, 46–49). The osteoblast/chondroblast-like cells initiate vascular calcification by releasing matrix vesicles (MV) (50) that are loaded with mineral crystals and are deposited in the ECM. Mineralized MVs have been detected in the calcified lesions of chronic kidney disease, atherosclerosis and diabetes. Furthermore, VSMCs cultured with high levels of phosphorus, calcium synthesize and secrete MVs harboring calcium, and phosphorus crystal-like structures (51, 52). MVs from osteoclast-like VSMCs and osteoblasts and hypertrophic chondrocytes have similar morphology and proteomics, which bind to collagen and induce apatite nucleation, resulting in endochondral and membranous osteogenesis (53–55). A recent study showed that hydroxyapatite nanocrystals reversibly induced osteogenic differentiation and calcification of VSMCs *in vitro*, and removal of the nanocrystals restored the original phenotype of the VSMCs and prevented calcification (33, 56). In a CKD rat model, inhibition of hydroxyapatite growth by sevelamer or pyrophosphate also slowed the progression of moderate to severe aortic calcification (57). Thus, osteoblast-like cells may also initiate vascular calcification through hydroxyapatite. Interestingly, the MVs from osteoblast-like cells express lower levels of calcification inhibitors like matrix gla protein (MGP) and fetuin A compared to normal VSMCs-derived MVs, whereas ALP, biomineralization-associated TNAP and phosphatidylserine (PS) are overexpressed in the former. TNAP hydrolyzes pyrophosphate, an inhibitor of vascular calcification, to produce inorganic phosphorus and PS that adhere to MV surface and adsorb calcium and phosphorus ions. Furthermore, the MVs secreted by osteoblast-like cells also express the annexins 2, 5, and 6, which function as calcium channels and allow ion influx (51, 58). Since MVs and exosomes aid intercellular communication, osteoblast-like cells may promote vascular calcification by secreting MVs that express aberrantly high levels of pro-calcification factors. Indeed, normal VSMCs co-cultured with osteoblast-like cell-derived MVs internalize the latter and undergo phenotypic transition characterized by the loss of contractile markers like smooth muscle 22 alpha (SM22a), and acquisition of the osteogenic markers (59). Microarray and bioinformatics analysis have identified significantly dysregulated non-coding RNAs in these MVs, which are enriched in signaling pathways closely related to osteogenic differentiation and calcification (60). Taken together, osteoblast-like cells in the vascular wall synthesize and secrete calcified MVs, which not only provide a site for calcium salt deposition but also initiate the phenotypic transition and calcification of adjacent cells in a paracrine manner.

## Origin of Osteoblast-Like Cells in Vascular Calcification

Although there are no osteoblast-like cells in the normal artery wall, several resident cells like VSMCs, vascular endothelial cells (VECs), pericytes, fibroblasts, calcified vascular cells (CVCs), mesenchymal stem cells (MSCs), or progenitor cells have the potential to differentiate or transdifferentiate into osteoblast-like cells in response to pathological stimuli (61).

## VSMCs and Osteoblast-Like Cells in Vascular Calcification

VSMCs are the main cell type in the arterial wall, and maintain vascular elasticity and contractility. Although VSMCs are terminally differentiated cells, they exhibit highly plastic phenotypes (62). The contractile VSMCs in the normal arterial wall are characterized by low growth, migration and protein synthesis, and express contractile markers such as SM22a, SMA, and SMM-HC. They can transdifferentiate into the synthetic phenotype that exhibits higher growth and migrations rates, produces more proteins, and expresses osteoblast and chondroblast markers (63). The calcified arterial lesions from humans and animal models also overexpress osteogenic and chondrogenic genes as a function of calcification degree, and have low levels of contractile markers. Furthermore, the osteogenic or chondrogenic markers are co-expressed with the VSMC contractile proteins, which indicates that VSMC can differentiate into osteogenic and chondrogenic cells as well (64–66). Consistent with this, osteoblast genes were upregulated and VSMC contractile genes were downregulated in human and animal VSMCs exposed to high levels calcium or phosphorus, which also formed massive alizarin red-staining calcium nodules. Likewise, hydroxyapatite and calcified elastin induced cultured VSMCs to lose the contractile phenotype and transdifferentiate into osteogenic cells, and removal of both restored the contractile phenotype and down-regulated bone-related genes, indicating that transdifferentiation of VSMCs is a reversible process (33). VSMCs expressing SM22α-Cre recombinase and Rosa26-LacZ Cre reporter alleles transdifferentiated into osteochondrogenic precursors and chondrocytes in MGP–/– or LDLr–/– and ApoE–/– mice, resulting in atherosclerotic medial and intimal calcification (67). Furthermore, calcium deposition and vascular calcification occur prior to the osteogenic phenotypic switch of VSMCs (42), and blocking the latter can inhibit or even reverse vascular calcification both *in vitro* and *in vivo*. There is evidence indicating that the different phenotypes of VSMCs have distinct embryonic origins (68, 69). It is possible that the contractile VSMCs first transdifferentiate into stem cells or other intermediate phenotypes prior to the osteoblast-like stage rather than differentiate directly. Thus, phenotypic reprogramming of VSMCs is a complex process and the regulatory pathways are largely unknown.

## VECs and Osteoblast-Like Cells in Vascular Calcification

VECs are derived from the mesoderm, and are the main cell type in the vascular intima. Under physiological conditions, VECs form a vascular barrier through tight junctions with endothelial-specific proteins. However, pathological stimuli can trigger endothelial-mesenchymal transition (EndMT) in the VECs, resulting in the acquisition of MSC-like multipotent differentiation capacity. Human aortic ECs cultured in hyperglycemic conditions express low levels of EC marker genes like CD31, and gain MSC markers like CD44 and CD10. Furthermore, the chondrocyte genes SOX9 and type II collagen, cartilage proteoglycan and calcium nodules are induced in VECs cultured in osteogenic media (70). Calcium nodules are also formed in MGP–/– human aortic ECs induced with high glucose and bone morphogenetic protein (BMP), as well as in human and bovine aortic ECs stimulated with BMP6 and oxidized low-density lipoprotein (LDL), or tumor necrosis factor-a (TNF-a) and interleukin-β (IL-β) (22, 71, 72). Furtherly, the research of Malhotra et al. shows EndMT and vascular calcification *in vitro* and *in vivo* are dependent upon BMP signaling in MGP-deficient mice, while the other hand, also suggests that activation of BMP signaling inhibits atherosclerosis in MGP-deficient mice fed a standard diet when compared to LDLR–/– mice fed a high fat diet, a murine model of atherosclerosis (73). These results indicate that VECs can transdifferentiate into osteoblast-like cells and promote vascular calcification. Mechanistically, BMP signal activation induces the phenotypic switch of VECs *via* EndMT. Consistent with this, calcified arteries from Mgp–/– and Ins2^Akita/+^ mice express CD31, multipotent markers like SOX2, Nanog and Oct3/4, and Runx2 and Osterix (74, 75). Interestingly, MGP–/– human VECs cultured with osteogenic medium or BMP2 show upregulation of SOX2, Nanog and Oct3/4 prior to that of Runx2 and Osterix, indicating that multipotent marker appear earlier in the endothelium. Lineage tracing in the Mgp–/– and Tie2-Gfp transgenic mice confirmed the localization of GFP-labeled ECs in calcified lesions, along with increased expression of Runx2 and Osterix, which further demonstrated that osteogenic cells can originate from VECs (74). In addition, VECs can also regulate vascular calcification in a paracrine manner by activating the VSMCs, MSCs, and fibroblasts. VSMCs and MSCs co-cultured with RANKL-expressing ECs or their conditioned medium transdifferentiated into osteoblast-like cells expressing Runx2 and ALP (76). Likewise, fibroblasts co-cultured with mechanically stimulated ECs or the conditioned medium also underwent calcification (77). Furthermore, the conditioned medium from inorganic phosphorus or indoxyl sulfate-induced umbilical vein ECs inhibited expression of osteopontin in MSCs and induced calcification in the presence of IL-8 (78). Finally, the MVs and exosomes secreted by aging or inflammatory factor-stimulated ECs express high amounts of calcium, BMP2, annexin and Notch3, and promote calcification of recipient cells following internalization (79).

## Pericytes and Osteoblast-Like Cells in Vascular Calcification

Pericytes are part of the microvascular system and closely related to endothelial cells. Recent studies show that pericytes or pericyte-like cells are abundant in the intima, media and adventitia of large, medium and small arteries, and can differentiate into osteoblasts, chondroblast-like cells and adipocytes. Furthermore, atherosclerotic calcified plaques of carotid and femoral arteries have significantly more pericytes compared to the non-calcified lesions, suggesting that these cells are involved in the formation of calcified plaques (80, 81). Consistent with the above, pericytes cultured with high content of advanced glycation end products, beta- glycerophosphate or glucocorticoid differentiate into osteoblast-like cells expressing Runx2, ALP, OC and other bone-related genes, and form calcium nodules. The latter may block MGP, an inhibitor of BMP4 and OPN, and activate the Axl signaling pathway (82–84). In addition, collagen glycosaminoglycan scaffolds loaded with pericytes gave rise to bone-like tissues in mice at the site of implantation, indicating that pericytes have spontaneous osteogenic potential (85, 86). Indeed, pericytes grown in normal medium can form multicellular nodules with a mineralized matrix containing extensive calcium salt deposits, and express high levels of Runx2 and the other osteogenic genes (21, 85). Therefore, pericytes have the potential to differentiate into osteoblast-like cells both spontaneously and in a stimuli-responsive manner, and contribute to mineralization.

## Fibroblasts and Osteoblast-Like Cells in Vascular Calcification

Fibroblasts are the major cell type located in the outer arterial membrane, and a potential source of osteoblast-like cells during vascular calcification. This is supported by the significant calcification seen in the aorta adventitia of high fat diet-fed ApoE–/– mice and individuals older than 60 years of age (87). Shao et al. found that transgenic mice expressing the osteoblast gene Msx2 in the adventitial cells developed extensive vascular calcification (88). Furthermore, primary fibroblasts cultured in the presence of recombinant transforming growth factor-1 (TGF-β) and beta-glycerophosphoric acid transformed into myofibroblasts and formed calcium nodules (89, 90). Lai et al. also confirmed that MSX2 expression levels increased in the myofibroblasts and subsequently contributed to calcification (91). Simionescu et al. found rat fibroblasts induced by elastin degradation products and/or TGF-β expressed high levels of Runx2, ALP, OC, and other bone-related genes, and formed calcium deposits that stained with the von Kossa dye (89). Likewise, Runx2 and OC were upregulated and calcium deposition was increased in the three dimensional cultures of Wistar rat fibroblasts after 2 weeks of exposure to high levels of inorganic phosphate or β-glycerophosphate (92). Therefore, adventitial fibroblasts are likely to transform into myofibroblasts and osteoblast-like cells to regulate vascular calcification.

## MSCs and Osteoblast-Like Cells in Vascular Calcification

MSCs are adult stem cells derived from the early mesoderm, and are characterized by high proliferation rates, self-renewal ability, multipotent differentiation potential, and immunosuppressive ability. Therefore, MSCs are the ideal biological tool for tissue repair and regeneration. The bone marrow-derived MSCs (BMSCs) differentiate into multiple cell types that are involved in cardiovascular pathologies, including vascular calcification. BMSCs transplanted into high cholesterol diet-fed rats with balloon injury in the abdominal aorta migrated to the BMP2-overexpressing arterial intima, and resulted in extensive calcification. Thus, MSCs can promote vascular calcification *via* the BMP2 signaling pathway (93). Kramann et al. implanted MSC-loaded collagen gels in the peritoneal cavity of rats with 5/6th nephrectomy or HFD-induced CKD, and detected Runx2, ALP, and sclerostin in the circulating MSCs, indicating osteogenic differentiation. Furthermore, a similar degree of calcification was observed after 8 weeks in the MSC-loaded gels as well as the arteries, both in terms of X-ray images and calcium content, indicating that MSCs contributed to vascular calcification after phenotypic conversion to osteoblast-like cells (94). Leszczynska et al. also confirmed the subcutaneous implantation of MSCs pretreated with chondrogenic medium gave rise to bone tissue in ApoE-/- and wild-type mice, and the extent of calcification was greater in the former (95). In addition, MSCs cultured in osteogenesis medium express osteogenic transcription factors and form calcium deposits *in vitro*. The MSCs isolated from ApoE–/– mice have greater osteogenic and chondrogenic capacity compared to wild-type cells, and is likely associated with the activation of uPAR-C5aR/Erk1/2/NF-kB axis (23). Wang et al. found that fluorescently labeled MSCs migrated to the calcified lesions in the HFD-fed LDLr–/– mice, and expressed osteogenic genes *via* TGF-β/SMAD2/4 signal activation (96). Interestingly, several groups have reported an inhibitory effect of MSCs on vascular calcification. For instance, intravenously injected MSCs significantly inhibited calcium and phosphorus deposition in the aortic walls, and also decreased serum phosphorus levels in the adenine-fed rat model of CKD (97). Similarly, conditioned medium from MSCs inhibited the osteogenic phenotypic switch and calcification of VSMCs even in the presence of β-glycerophosphate, and this effect was reversed by recombinant BMP2 *via* activation of the SMAD1/5/8 pathway. In addition, MSCs reduced calcification of co-cultured VSMCs in the osteogenic medium by inactivating the Wnt5a/β-catenin pathway (98). Therefore, the role of MSCs in vascular calcification is highly complex, and may depend on the environmental cues.

## Progenitor Cells and Osteoblast-Like Cells in Vascular Calcification

Progenitor cells are precursors with multipotent differentiation potential. Studies increasingly show that both endothelial and VSMC progenitors are closely related to arterial calcification. For instance, circulating endothelial progenitor cells expressing the late osteoblast differentiation marker osteocalcin (OC) are abundant in patients with diabetes, CKD and coronary atherosclerosis. Furthermore, the number of OC+ circulating endothelial progenitor cells correlates positively with the calcium score and spotty calcification in coronary artery disease and hemodialysis patients (99, 100). Endothelial progenitors isolated from the peripheral blood of hemodialysis patients showed significantly increased calcium deposition *in vitro*. However, adding vitamin D receptor activator into medium of serum from uremia patients would inhibit calcification of endothelial progenitor cells (99). Similarly, the number of circulating OC+ endothelial progenitor cells in postmenopausal women and hemodialysis patients decreased significantly by administering bisphosphonates and vitamin D receptor activator, which also reduced vascular calcification (20). Taken together, OC+ endothelial progenitor cells can differentiate into osteoblast-like cells and promote vascular calcification. The CD10+ human perivascular progenitor cells, also known as adventitial cells due to their anatomical location, also show osteogenic potential. CD10 is significantly upregulated in the calcified vs. normal arteries. The CD10+ progenitor cells isolated from human abdominal white adipose tissue and placenta expressed osteoblastic transcription factors and other proteins in an NF-κB-dependent manner when cultured in osteogenic medium for 28 days, and formed mineralized nodules. Silencing the CD10 gene significantly decreased the bone forming ability of these progenitors (101). Kramann et al. detected abundant Gli1+ VSMC progenitors in the arterial adventitia, media and intima of mice with acute femoral artery injury or CKD, which promoted tissue repair (102). Furthermore, genetic fate tracing indicated that Gli1+ progenitor cells migrated from the adventitia into the intima and media of ApoE–/– mice with CKD, and expressed osteogenic genes that promoted arterial calcification. Furthermore, knocking down Gli1 inhibited arterial calcification (102). Cho et al. discovered Sca-1+/PDGFRα- progenitor cells in the bone marrow and blood vessel walls that differentiated into both osteoblasts and osteoclasts. Subcutaneous transplantation of Sca-1+/PDGFRα- progenitor cells and bone matrix in C57 mice resulted in a highly mineralized bone-like structure after 8 weeks. However, simultaneous injection of PPARγ significantly decreased calcification volume and calcium scores by inducing the differentiation of Sca-1+/PDGFRα- progenitor cells into osteoclasts. Consistent with the above, Sca-1+/PDGFRα- progenitor cells also aggravated arterial calcification in atherosclerotic ApoE–/– mice, which was reversed by PPARγ (39, 103).

## CVCs and Osteoblast-Like Cells in Vascular Calcification

CVCs are a subset of VSMCs that characteristically express a modified form of ganglioside sialyllactose ceramide, along with osteogenic and chondrogenic markers. The CVCs can form calcium nodules following stimulation with transforming growth factor-β or 25-hydroxycholesterol. Similarly, mechanical stimulation or cAMP treatment also promotes osteogenic differentiation and mineralization of the CVCs. Overexpression of liver X receptor enhanced CVC mineralization by increasing fatty acid synthesis and lipid accumulation. In addition, subcutaneously transplanted CVCs formed calcium nodules akin to calcified atherosclerotic plaques in the Apoe–/– mice (104). Mechanistically, osteogenic differentiation and calcification of CVCs is mediated by the heat shock protein (HSP) 70, which binds to MGP and activates the BMP2/P-SMAD1/3/5 pathway (105). In addition, macrophages stimulated with ultrafine particles or their conditioned media induced osteogenic differentiation and mineralization of CVCs by partially activating the NF-κB signaling pathway (106). Conversely, the spontaneous differentiation and calcification of CVCs were inhibited by the protein kinase A-specific inhibitor KT5720, or insulin-like growth factor I, which are known to activate the ERK and PI3K pathways (107). Abedin et al. found that activation of p38-MAPK and PPARγ pathways decreased both spontaneous and IL-6-mediated osteogenesis of CVCs (108). These results indicate that CVCs can not only form calcium nodules spontaneously, but also be induced to exacerbate vascular calcification.

## Origin and Function of Osteoclast-Like Cells in Vascular Calcification

Bone formation depends on the balance between the osteoblasts and osteoclasts. The osteoclasts mediate bone mineral matrix resorption and prevent excessive bone mineralization by producing various enzymes. While excessive bone resorption by osteoclast activity leads to osteoporosis, aberrant bone formation by hyperactive osteoblasts result in osteopetrosis. Osteoblast-like cells are abundant in calcified blood vessels and promotes vascular calcification, although the role of osteoclast-like cells are poorly understood.

Osteoclast-like cells have been detected in both atherosclerotic plaques and calcified medial layer, although the number is small and mainly concentrated in the heavily calcified lesions. Qiao et al. found osteoclast-like cells in human carotid plaques (38). Han et al. analyzed the calcified media and intima of 282 lower extremity artery samples, and detected bone tissue and osteoclasts in some heavily calcified regions (109). Similarly, tartrate-resistant acid phosphatase (TRAP), an osteoclast-specific marker gene, was also detected in the calcified vessels of hypertensive patients (37). Carbonic anhydrase, the key enzyme involved in osteoclast-mediated bone resorption, is significantly up-regulated in the atherosclerotic plaques of carotid, femoral and aortic vessels, and co-expressed with TRAP (110). These findings indicate that osteoclast-like cells regulate atherosclerosis and vascular calcification, although the mechanisms remain unknown. Monocytes and macrophages can differentiate into osteoclasts in the presence of inflammatory cytokines such as IL-6, IL-1, and TNF-α *via* activation of the RANKL/Akt signaling pathway. Osteoclastogenic differentiation of these cells is inhibited by oxidized low density lipoprotein or high levels of inorganic phosphate (111, 112). In the atherogenic ApoE–/– mice as well, macrophages transdifferentiate into osteoclasts *via* the Runx2/RANKL axis. Consistent with this, macrophages derived from human peripheral blood mononuclear cells differentiate into osteoclasts expressing cathepsin K and TRAP, and their calcium resorption activity is controlled by N-acetylglucosamine-1-phosphate transferase alpha and beta subunits (113). In addition, macrophages can effectively decalcify ectopic deposits by overexpressing carbonic anhydrase 2, which is silenced in mice with medial calcification in small arteries (114). Vascular progenitor cells also differentiate into osteoclast-like cells in the calcified lesions. The Sca-1+/PDGFRa- progenitor cells from mouse aorta differentiate into functional osteoclast-like cells *in vitro* in the presence of suitable factors, indicating their bipotent differentiation potential. In addition, Sca-1+/PDGFRa- cells implanted subcutaneously in C57 mice and atherogenic ApoE–/– mice differentiated into osteoclast-like cells in the presence of PPARγ, resulting in reduced calcium scores and calcification volume, suggesting a suitable therapeutic strategy for vascular calcification (39). Co-culture of calcified elastin and bone-marrow-derived osteoclast-like cells decreased significantly calcium content of the calcified elastin without elastin degradation and subcutaneous transplantation in rat of the mixture containing pure aortic elastin and osteoclast-like cells limited elastin calcification compared to control group (40). Bas et al. and Qiao et al. also found that osteoclast-like cells and macrophages inhibited and reversed calcification through mineral resorption (32, 38). However, the exact role of osteoclast-like cells in vascular calcification is largely ambiguous. RNA-seq analysis of rabbit atherosclerotic plaques showed enrichment of genes and KEGG pathways related to osteoclast differentiation, which have a bidirectional impact on osteoblast differentiation (115). In another study, knocking out Runx2 in VSMCs decreased RANKL expression, osteoclast numbers and the severity of calcification. Furthermore, VSMCs co-cultured with RANKL-overexpressing bone marrow-derived macrophages differentiated into osteoclast-like cells and increased vascular calcification (116). Thus, the role of osteoclast-like cells in vascular calcification needs further clarification.

## The Potential of Cell-Based Therapeutic Strategies for Vascular Calcification

Over the years, great progress has been obtained about the pathogenesis of vascular calcification, but the treatment strategies for vascular calcification are limited. As discussion above, osteoblast-like cells play an important role in vascular calcification. Owning the role of osteoblast-like cells, many researches showed inhibition of osteogenic differentiation of VSMCs or recovery of contractile phenotype of VSMCs ameliorated vascular calcification both *in vitro* and *in vivo* (117–119). Lin et al. also reported SMC-specific Runx2 knockout significantly reduced vascular osteochondrogenesis and calcification in mouse (10). These results suggest it is possible to treat vascular calcification at cellular level by regulating phenotype changes of VSMCs. In addition, owing mineral resorption capacity of osteoclast-like cells, osteoclasts efficiently remove deposited minerals from calcified elastin both *in vitro* and *in vivo* (40). Co-culture of macrophages and VSMCs, inhibition of macrophages differentiated into osteoclasts contributed to VSMCs calcification. Furthermore, Barinda et al. found also macrophages reversed ectopic calcification in extracellular matrix through overexpressing carbonic anhydrase 2, which mediates hydrogen ions formation inducing mineral dissolution (114). In a mouse model of ectopic calcification, promoting the differentiation of Sca-1(+)/PDGFRα (-) progenitor cells with a PPARγ agonist into osteoclast-like cells attenuated ectopic calcification severity (39). These studies indicate osteoclast-like cells—and macrophage-based vascular calcification cell therapy has potential. However, Gbaguidi et al. reported macrophages near calcium deposits were deficient in RANKL-RANK axis and cathepsin K hindering their ability to reabsorb minerals in human atherosclerotic plaques (120). Recently, MSC therapy has been developed in many areas, which is a new strategy for treatment of vascular calcification. Calcified VSMCs induced with β-GP were cultured with conditioned media from MSCs and mineral deposition in VSMCs was inhibited, which was associated with inactivation of the BMP2–Smad1/5/8 signaling pathway and suppression of osteogenic phenotype, inflammation, and apoptosis (97, 98). Exosomes from MSCs were used to culture VSMCs and inhibited high phosphorus-induced VSMCs calcification (121). To similar these studies, indirect co-culture of VSMCs and MSCs in a transwell system reduced also VSMCs calcification involving in inhibiting of wnt signaling pathways (122). These researches suggest MSCs regulate vascular calcification in a paracrine manner. *In vivo*, adipose-derived MSCs were injected into a rat model of chronic kidney disease, which inhibited the progression of vascular calcification (123). However, some studies also reported MSCs have the potential of osteogenic differentiation (124). BMSCs transplantation promoted vascular calcification in hyperlipidemic rats subjected balloon injury and MSCs were implanted in c57BL/6 mice and Apoe–/– mice can form bone (93, 95). Taken together, cell treatment for vascular calcification based on osteoclast-like cells or macrophages and even MSCs is possible, but there are still some controversies and more research is needed.

## Future Research

Although the potential sources of osteoblast-like cells in calcified lesions have been identified, the transdifferentiation of vascular cells into osteoblast-like cells is more complex and needs further research. Different subsets of VSMCs and ECs had been identified in vascular walls, and the VSMCs located in different segments of the aorta originate from different embryo layers. The obvious question herein is whether specific VSMC and ECs subsets are wired to differentiate into osteoblast-like cells. Both VSMCs and ECs undergo a phenotypic trajectory during transdifferentiation into osteoblast-like cells. The underlying molecular mechanisms remain to be elucidated, especially during early phenotypic transition. Limited number of osteoclast-like cells have also been identified in calcified arteries, especially in areas of severe calcification. Recent studies show that osteoclast-like cells promote atherosclerotic calcification in Apoe–/– mice and rabbits, although others have correlated increased number of osteoclast-like cells with atherosclerotic decalcification. Therefore, the exact role and origin of these cells are ambiguous, and have to be elucidated. Osteoclasts have bone resorptive activity, and an imbalance between osteoblastogenesis and osteoclastogenesis is present in vascular calcification. It remains to be seen whether osteoclasts or osteoclast-like cells are a promising therapeutic strategy for vascular calcification, and the most pressing question in this regard is to optimize the differentiation of vascular wall cells into osteoclast-like cells.

## Conclusions

**Figure 1:**
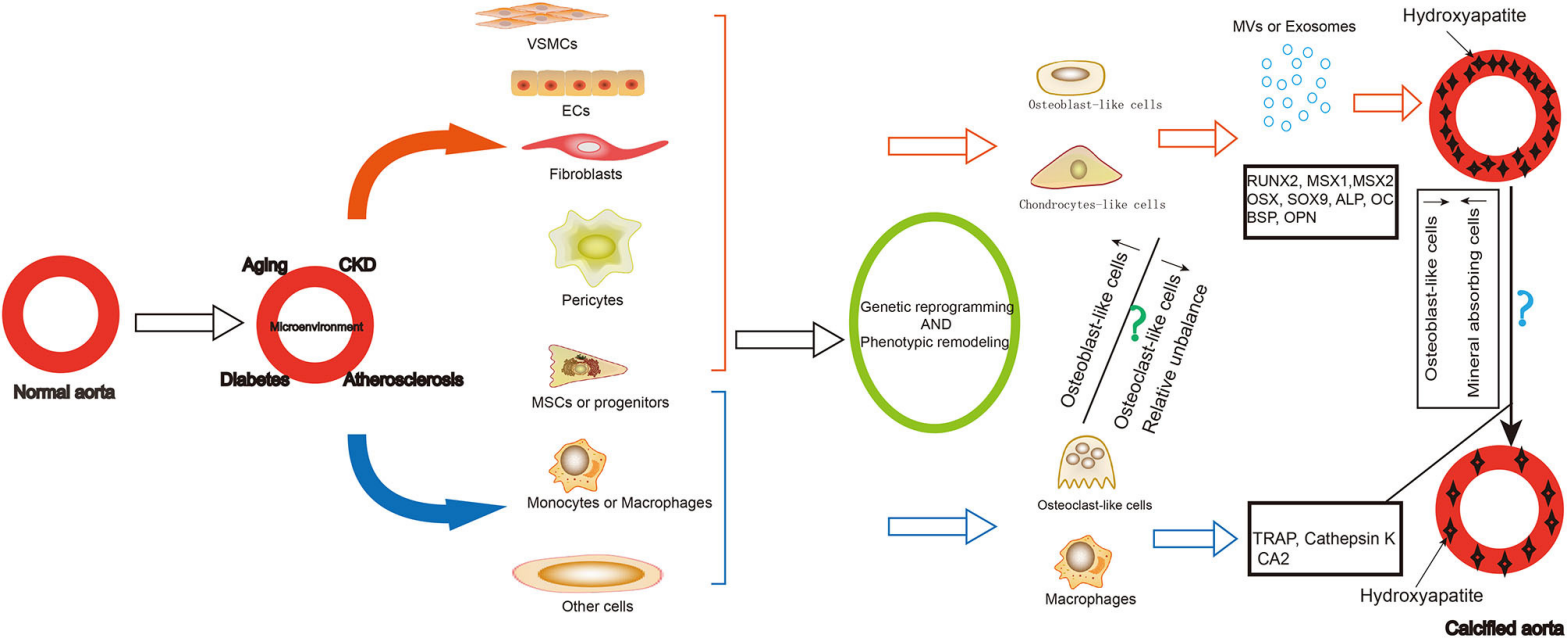
Summary of the origin and role of osteoblast-like cells and osteoclast-like-cells in vascular calcification. Vascular calcification is common in older adults, patients with chronic kidney disease, diabetes, and atherosclerosis. During the formation of vascular calcification, many cells differentiated into osteoblast-like cells or chondrocytes-like cells, including VSMCs, ECs, fibroblasts, pericytes, MSCs and progenitors. They express osteoblastic differentiation transcription factors and bone matrix proteins as well as secrete exosomes and matrix vesicles contributing to the development and progression of vascular calcification. Some cells can also differentiate into osteoclast—like cells, including some MSCs or progenitors, macrophages. They play a role in demineralization of the vascular calcification. The reduction of osteogenic phenotype cells and the increase of mineral reabsorption cells may be a strategy for vascular calcification cell therapy. VSMCs, vascular smooth muscle cells; ECs, endothelial cells; MSCs, mesenchymal stem cells; MV, matrix vesicles; RUNX2, RUNX family transcription factor 2; MSX, msh homeobox; SOX9, SRY-related high mobility group-box gene9; OSX, Sp7 transcription factor; ALP, alkaline phosphatase; OC, osteocalcin; BSP, bone sialoprotein; OPN, osteopontin; TRAP, acid phosphatase 5, tartrate resistant; CA2, carbonic anhydrase 2.

Vascular calcification is a cell-mediated, reversible and active regulated process resembled to bone mineralization. Osteoblast-like cells and limited number of osteoclast-like cells have been identified in calcified lesions (Figure 1). While the former promote calcification of the arterial walls, the latter have a demineralizing effect. Therefore, tracing the origin of these cells and the trajectory of their phenotypic remodeling, along with identification of core regulatory molecules, can further elucidate the mechanism of vascular calcification and reveal new therapeutic targets.

## Author Contributions

WJ, ZZ, YL, CC, HY, QL, and MH: literature search and analysis. WJ: writing the manuscript. XQ and WJ: review and revise the manuscript. XQ: the funding acquisition. All authors contributed to the article and approved the submitted version.

## Conflict of Interest

The authors declare that the research was conducted in the absence of any commercial or financial relationships that could be construed as a potential conflict of interest.
